# Role of hypoxia-induced exosomes in tumor biology

**DOI:** 10.1186/s12943-018-0869-y

**Published:** 2018-08-11

**Authors:** Chuchu Shao, Fengming Yang, Suyu Miao, Weitao Liu, Chaoshan Wang, Yongqian Shu, Hua Shen

**Affiliations:** 1 Department of Oncology, The Affiliated Sir Run Run Hospital of Nanjing Medical University, Nanjing, China; 2 Department of Oncology, the First Affiliated Hospital of Nanjing Medical University, 300 Guangzhou Road, Nanjing, 210029 People’s Republic of China; 3 Department of Breast Surgery, The First Affiliated Hospital of Nanjing Medical University, Nanjing, China; 4 Department of Pathology, Nanjing Medical University, Nanjing, China

**Keywords:** Exosome, Extracellular vesicles, Hypoxia, Cancer

## Abstract

**Purpose:**

Hypoxia is a major regulator of angiogenesis and always influences the release of exosomes in various types of tumors. The present review aimed to assess the role of hypoxia-induced exosomes in the tumor biology.

**Methods:**

The relevant publications were retrieved from PubMed using keywords such as hypoxia, exosome, extracellular vesicles, tumor, cancer, and other similar terms.

**Results:**

Recent studies have shown that cancer cells produce more exosomes under hypoxic conditions than do parental cells under normoxic conditions. The secretion and function of exosomes could be influenced by hypoxia in various types of cancer. Hypoxia-induced exosomes play critical roles in tumor angiogenesis, invasion, metastasis, and the immune system.

**Conclusions:**

These findings provide new insights into the complex networks underlying cellular and genomic regulation in response to hypoxia and might provide novel and specific targets for future therapies.

## Background

Hypoxia, the condition of insufficient oxygen, is a common feature of malignant tumors. In a majority of malignancies, the median oxygen level is about 10 mmHg, while the normal tissues have rather high oxygen pressure (between 40 and 60 mmHg) [1]. This phenomenon is attributed to the high oxygen demand from proliferating cancer cells and low oxygen supply due to irregularities in tumor vascularization or distance from supporting blood vessels [2, 3]. Hypoxia has been well acknowledged as an intricate element of the tumor microenvironment involved in tumor aggressiveness and metastasis [4–6]. In response to hypoxia, the cancer cells alter the transcription of numerous genes in conjunction with the oxygen-monitoring mechanism, including hypoxia-inducible factors (HIFs), the major components of hypoxia signaling pathways [7].

HIFs are dimeric proteins that comprise of an oxygen-sensitive subunit HIF-α (HIF-1α, HIF-2α, or HIF-3α) and a constitutively expressed HIF-1β subunit [8, 9]. In the presence of oxygen, HIF-1α is hydroxylated by prolylhydroxylase (PHD), following which, the hydroxylated HIF-1α is recognized by von Hippel-Lindau (VHL). This serves as the targeting subunit of an E3 ubiquitin ligase complex and thereby tags HIF-1α for ubiquitination and degradation by the 26*S* proteasome [10]. However, under hypoxic conditions, PHDs are no longer active to hydroxylate HIF-1α, resulting in HIF-1α stabilization and dimerization with HIF-1β. The expression of HIF-1α is also influenced by another oxygen sensor factor-inhibiting HIF-1α (FIH). As a key regulator of HIF-1α, FIH-1 catalyzes an asparagine hydroxylation step that controls the association of HIF-1α transcription factors with CBP/p300 transcriptional co-activators and reduces the transcriptional activity of HIF-1α [11]. Given the observations that most malignant tumors experience hypoxic conditions, HIFs activation occurs in almost all types of cancer. A large part of HIF-dependent hypoxic response relies on intercellular signalling, which regulates the expression of genes associated with angiogenesis, epithelial-to-mesenchymal transition (EMT), metastasis to promote cell survival and the adaptation of cells to hypoxic conditions [12]. In addition to intercellular hypoxic signaling pathways, recent studies have shown the importance of the crosstalk between tumor cells and their microenvironmental factors via extracellular vesicles (EVs)s secreted from hypoxic tumor cells [13].

EVs are cell-derived vesicles with different sizes and intracellular origins, which can be characterized into three categories: exosomes (30–100 nm diameter), microvesicles (MVs) (100–1000 nm diameter), and larger vesicles termed oncosomes (1–10 μm diameter) [14–17]. Recently, the role of EVs, especially exosomes secreted by tumor cells in modulating cell-to-cell communication has been highlighted [18, 19]. Exosomes are generated from the inward budding of late endosomes, and thus, released into the extracellular space upon fusion with the plasma membrane [20, 21]. Once released into the extracellular space, exosomes can reach the recipient cells and deliver the contents to elicit the functional responses and promote phenotypic changes that would affect the physiological or pathological status [22]. The contents of exosomes are complex, including various types of proteins, RNAs, and DNAs that can act as messengers for cell communication in local and distant microenvironments [23–25]. RNAs are reported as the major bioactive factors of tumor cell-derived exosomes, along with several species of non-coding RNAs including microRNAs (miRNAs), long non-coding RNAs (lncRNAs), and circular RNAs [26–28]. These functional non-coding RNAs delivered by exosomes to recipient cells can regulate numerous gene expression to promote tumor growth, local invasion, and create premetastatic or metastatic niches.

It is now clearly evident that exosomes derived from tumor cells play critical roles in modulating the tumor microenvironment [13]. Recent findings have reported that hypoxia stimulate increased levels of exosomes, thereby facilitating tumor intercellular communication at a distance, indicating a role of exosomes as vital regulators in hypoxic tumors [29, 30]. In breast cancer, the cancer cells exposed to hypoxia has been reported increase their production of exosomes in an HIF-dependent manner, which stimulate invasion and metastasis by contacting with recipient cancer cells [31]. In the present review, we will discuss how exosomes induced by hypoxia participate in tumor angiogenesis, invasion, metastasis, and immune system.

### Hypoxia induces the release of exosomes

Exosomes are vital mediators of intercellular communication that can transfer the cells’ phenotype to non-hypoxic cells through the production of exosomes. As mentioned above, recent researches indicated that hypoxia can induce the release of exosomes. Target genes include numerous plasma membrane receptors such as glucose transporter (GLUT-1), epidermal growth factor receptor (EGFR), transfer receptors, P-glycoprotein (P-gp), and multidrug resistance protein 1 (MRP1). The altered receptor expression can increase the receptor activation and internalization or result in receptor clustering, which consequently induces endocytosis and promotes exosome release [32]. Interestingly, the small GTPases, RAB27A and RAB27B, were implicated in exosome secretion in human HeLa cells [33, 34]. In breast cancer, RAB22A was also required for mediating the formation of extracellular vesicles [31]. However, the specific molecular mechanisms regulating the exosome secretion are yet to be elucidated.

In addition to the quantitative impact of exosome secretion, hypoxia stress also causes significant changes in the content and function of exosomes. As Kore et al. found that hypoxic exosomes derived from GBM cells selectively elevated some proteins such as protein-lysine 6-oxidase (LOX), thrombospondin-1 (TSP1) and vascular derived endothelial factor (VEGF), which were known to be associated with tumor progression, metastasis and angiogenesis [35]. Moreover, several pieces of evidence have established that hypoxia regulates the expression of different non-coding RNAs delivered by exosomes. For example, miR-210 is a well-established target of HIF-1α and strongly induced in most cancer types in response to hypoxia. King et al. demonstrated that breast cancer cells release high levels of exosomes and miR-210 in hypoxic exosomes in an HIF-1α-dependent manner. Furthermore, three different breast cancer cell lines were exposed to moderate (1% O_2_) and severe (0.1% O_2_) hypoxia, leading to significant increase in the number of exosomes [36]. Similarly, in another study addressing the molecular mechanisms regulating exosomal shedding, Umezu et al. found that hypoxia-resistant multiple myeloma (HR-MM) cells produced more exosomes with a significantly higher expression of miR-135b as compared to normoxic cells, indicating that the tumor-secreted exosomes could be induced by hypoxia [37].

### Hypoxia influence the exosomes secreted by the tumor microenvironment

Most tumors develop a hostile tumor microenvironment associated with the expansion of hypoxic and necrotic areas as the existing vasculature cannot fulfill the increasing oxygen demand of rapidly expanding tumors. The tumor microenvironment can be subdivided into the chemical microenvironment (for example, low oxygen, low pH, and low nutrition), the diverse extracellular signaling molecules, and the cellular microenvironment, which include tumor cells, stromal cells, extracellular matrix (ECM), and inflammatory immune cells [38]. The immune component of the tumor microenvironment is comprised of myeloid cells including tumor-associated macrophages (TAMs), myeloid-derived suppressor cells (MDSCs), dendritic cells (DCs), and tumor-infiltrating lymphoid cells (TILs). All these cells are greatly affected by hypoxic stress present in the tumor microenvironment. Hypoxia can interfere with the differentiation and function of immune cells by modulating the expression of co-stimulatory receptors and the type of cytokines produced by these cells. Furthermore, exosomes secreted by other cells also could be influenced by hypoxia. For example, Fernanda et al. revealed that the TGF-β1-containing exosomes from injured epithelial cells activate the fibroblasts that in turn, initiate tissue regenerative responses and fibrosis. Thus, the present study suggested that TGF-β1 mRNA transported by exosomes constitute a rapid response initiating the tissue repair/regenerative responses and the activation of fibroblasts when resident parenchyma is injured [39].

The content of hypoxia-induced exosomes varies depending on the cell origin including signal transducers, transcription factors, enzyme, lipids, mRNAs, and non-coding RNAs. In different cancers, exosome-mediated signaling promotes tumor progression through communication between the tumor and surrounding stromal tissues, involving tumor angiogenesis, invasion, metastasis, and immune escape. The function of exosome-mediated signaling pathways under hypoxic states will be discussed in the following sections.

### Hypoxia-induced exosomes enhance cancer angiogenesis

Angiogenesis is a complex process involving the sprouting and configuring of new MVs from pre-existing blood vessels [40, 41]. It is a critical step in cancer progression via stimulation of the tumor growth. This process is highly regulated by a group of ligands and receptors through multiple signaling pathways. The proangiogenic signaling molecule vascular endothelial growth factor (VEGF) and its cognate receptor (VEGFR) play a central role in angiogenesis and are highly expressed in tumor tissues [42]. VEGF signaling stimulates cellular pathways that lead to the formation and branching of new tumor blood vessels, facilitating rapid tumor growth, and metastatic potential [43]. The development of antiangiogenic treatments by several investigators is focused on inhibiting the VEGF/VEGFR signaling.

**Fig. 1:**
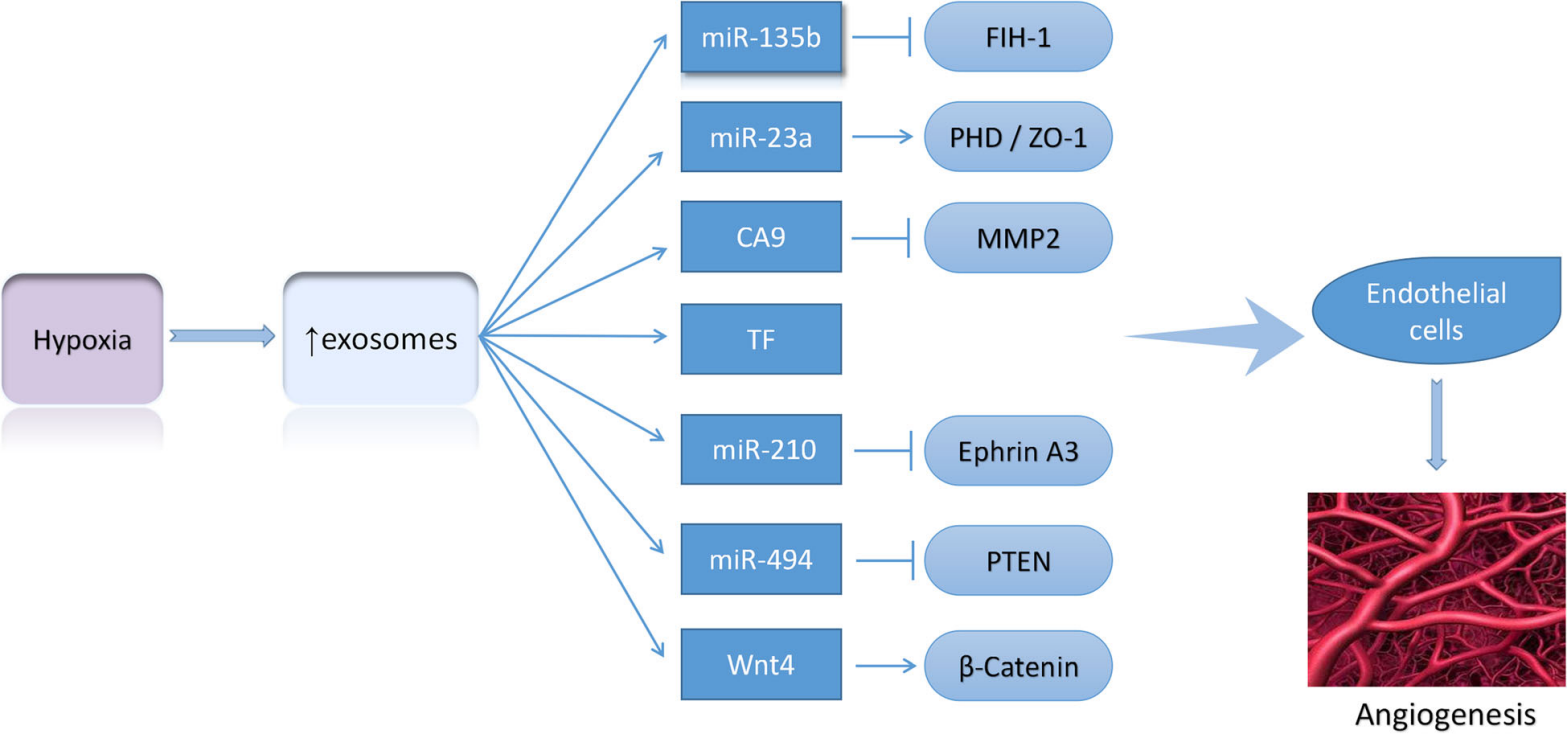
Hypoxic-induced exosomes promote angiogenesis in multiple cancers. Under chronic hypoxic conditions, cancer cells secrete higher levels of exosomes. The upregulated exosomal ncRNAs and specific proteins induced by hypoxia are taken up by the surrounding endothelial cells, resulting in the accelerated angiogenesis

Recent studies have highlighted the role of hypoxia-induced exosomes in angiogenesis and tumor development. For example, exosomal miR-135b has also been found to be transferred into endothelial cells by hypoxia-resistant multiple myeloma (HR-MM) cells and target HIF-1, thereby enhancing angiogenesis [37]. Exosomes isolated from hypoxic lung cancer cells contained miR-23a, which increased the angiogenesis by targeting prolyl hydroxylase and tight junction protein ZO-1 [44]. Also, Tadokoro et al. reported that exosomes derived from hypoxic leukemia cells enhanced the tube formation in human umbilical vein endothelial cells (HUVECs) via miR-210 [45]. Moreover, Mao et al. revealed that hypoxia-induced miR-494 promotes angiogenesis in non-small cell lung cancer (NSCLC). Firstly, hypoxia induces the expression of miR-494 via the HIF-1α-mediated mechanism. The upregulated miR-494 was secreted from tumor cells into microenvironment and delivered into ECs via exosomes, followed by downregulation of PTEN and activated Akt/eNOS pathway in ECs; consequently, the tumor development is exacerbated by promoting angiogenesis [46] (Fig. 1).

**Table 1:** Hypoxia-induced exosomes involved in cancer biology

Regulatory factors	Cancer types	Response to hypoxia	Biological function	Mechanism	Ref
Exosomal miR-135b	Multiple myeloma	Increased	Increase angiogenesis	Downregulates its target FIH-1	[37]
Exosomal miR-23a	Lung cancer	Increased	Increase angiogenesis and migration	Inhibition of PHD1, PHD2 and ZO-1	[44]
Exosomal miR-210	Leukemia	Increased	Increase angiogenesis	Inhibits the expression of Ephrin A3	[45]
Exosomal miR-494	Non-small cell lung cancer	Increased	Increase angiogenesis	Downregulates PTEN and activates Akt/eNOS pathway in ECs	[46]
Exosomal miR-21	Oral squamous cell carcinoma	Increased	Promote migration and invasion	Downregulate a pool of genes and induces EMT of these cells.	[56]
Exosomal miR-940	Epithelial ovarian cancer	Increased	Regulate immune response	Induces macrophages to express higher levels of the M2-type markers CD163 and CD206	[70]
Exosomal miR24-3p	Nasopharyngeal carcinoma	Increased	Regulate immune response	Not mentioned	[71]
Exosomal miR-210	Hypoxic cancer	Increased	Increase angiogenesis	Inhibits the expression of Ephrin A3 and PTP1B	[72]
Exosomal linc-UCA1	Bladder cancer	Increased	Promote migration and invasion	Promotes EMT	[55]
Exosomal linc-RoR	Hepatocellular cancer	Increased	Promote migration and invasion	Not mentioned	[73]
Exosomal proteins	Prostate cancer	Increased	Promote migration and invasion	Not mentioned	[54]
Exosomal CA9	Renal cell carcinoma	Increased	Increase angiogenesis	Upregulates its target MMP2	[74]
Exosomal TF	Glioblastoma multiforme	Increased	Increase angiogenesis	Enhances TF/VIIa-mediated PAR-2 activation and activates endothelial cells	[75]
Exosomal Wnt4	Colorectal cancer	Increased	Increase angiogenesis	Increases β-catenin nuclear translocation in endothelial cells	[76]
Exosomal MMP13	Nasopharyngeal carcinoma	Increased	Promote migration and invasion	Not mentioned	[77]
Exosomal HIF1α	Nasopharyngeal carcinoma	Increased	Promote migration and invasion	Promotes EMT	[78]
Exosomal TGF-β1 and miR23a	Hypoxic cancer	Increased	Regulate immune response	TGF-β1 downregulates NKG2D and miR23a directly targets CD107a	[79]

### Hypoxia-induced exosomes promote cancer cell invasion and metastasis

Invasion and metastasis are the determination features of malignant tumors. It is well-known that tumor invasion and metastasis involve multiple steps, among which, the epithelial-mesenchymal transition (EMT) is an absolute and crucial step for metastasis [32]. During EMT, the cancer cells shed their epithelial features, remodel their cytoskeleton, and acquire a mesenchymal phenotype that correlates to enhanced migratory and invasive capacity [47]. At the molecular level, the changes occurring during EMT are explained by the loss of epithelial and the gain of mesenchymal markers. The loss or downregulation of E-cadherin is a major event in EMT, and it can be identified as one of EMT biomarkers [48]. It is known as a transmembrane glycoprotein expressed in epithelial cells that regulate cell-to-cell contact, cell shape, and polarity [49]. Moreover, it connects the adjacent cells through homophilic interactions as well as linked to the cytoskeleton via a multi-catenin complex that is attached to their cytoplasmic tails [50, 51]. In this complex, β-catenin and p120 are directly associated with E-cadherin, while α-catenin is the link between β-catenin and the actin microfilament network of the cytoskeleton [52]. Loss of E-cadherin results in the loss of cell-to-cell contact, disruption of E-cadherin-catenin complex, abnormal activation of β-catenin signaling, and cellular cytoskeletal alterations. Overall, these changes are essential for the cells to lose their epithelial polarity and acquire an invasive phenotype [53].

An increasing number of studies provide novel evidence that under hypoxic conditions, the migration and invasion ability of cancer cells can be enhanced by hypoxia-induced exosomes. Ramteke et al. reported that hypoxia-induced exosomes promoted the invasiveness and metastasis of prostate cancer cells by targeting adherens junction molecules [54]. In the study, hypoxia-induced exosomes promoted the loss of E-cadherin in PC3 cells along with an increase in cytoplasmic and nuclear β-catenin expression, which might be responsible for the observed increase in the invasiveness, motility, and stemness of PCA cells by TDEs. Consecutively, another study also found that hypoxia enhances the exosome-mediated shuttling of lncRNA-UCA1 into bladder cancer cells, and hypoxic exosomal lncRNA-UCA1 also promotes development, invasion, and migration of tumor cells in vitro and in vivo [55]*.* Furthermore, Ling et al. showed that exosomes from oral squamous cell carcinoma (OSCC) cells were upregulated under hypoxia, and the upregulated exosomal miR-21 downregulates a pool of genes and induces EMT of the target normoxic cells [56]. Other studies provide more evidence on the role of hypoxic-induced exosomes in tumor invasion and metastasis as below (Table 1).

### Hypoxia-induced exosomes influence cancer immune system

Reduced immune surveillance is a key mechanism through which primary tumors create permissive environments in secondary organs that favor the development of metastasis [57–59]. Accumulating evidence has shown that tumor-derived exosomes induce T-cell apoptosis, reduce NK cells activity, inhibit IFN-γ dependent class II expression of macrophages, and alter the differentiation of monocytes to increase the myeloid-derived suppressor cell (MSDCs) population, which leads to a collective failure of the immune system in containing the cancer growth [60–64]. For example, SW et al. found that breast cancer exosomes directly suppress the T-cell proliferation and inhibit the NK cell cytotoxicity, and hence suppressed the anticancer immune response in premetastatic organs [65]. MiRNAs in lung cancer cell-derived exosomes can silence the transcripts associated with Toll-like receptor (TLR) family in macrophages. This mechanism stimulates the macrophages to secrete proinflammatory cytokines, which supports enhanced tumor dissemination. Cancer cells are capable of inhibiting the anti-tumor functions of the host immune system via an exosome-induced signaling mechanism [66]. Moreover, T lymphocytes are not the sole immune cells targeted by tumor-derived exosomes. The activities of human NK cells, B-cells, and monocytes are impaired by co-incubation in the presence of exosomes. In NK cells, the downregulation of the expression of the activating receptors, especially NKG2D, is induced by tumor-derived exosomes carrying the MICA and MICB ligands [67]. NK-cell activation and cytotoxicity are inhibited by TGF-β, which is predominantly displayed on exosomes as TGF-latency associated protein (TGF-LAP). Moreover, tumor-derived exosomes can synthesize adenosine from ATP by virtue of carrying CD39 and CD73, which are implicated in inducing suppressive activity in activated B cells as adenosine can convert the activated B-cells into regulatory B-cells [68]. In addition, tumor-derived exosomes skewed the differentiation of myeloid precursor cells towards development into highly suppressive MDSCs [69].

**Fig. 2:**
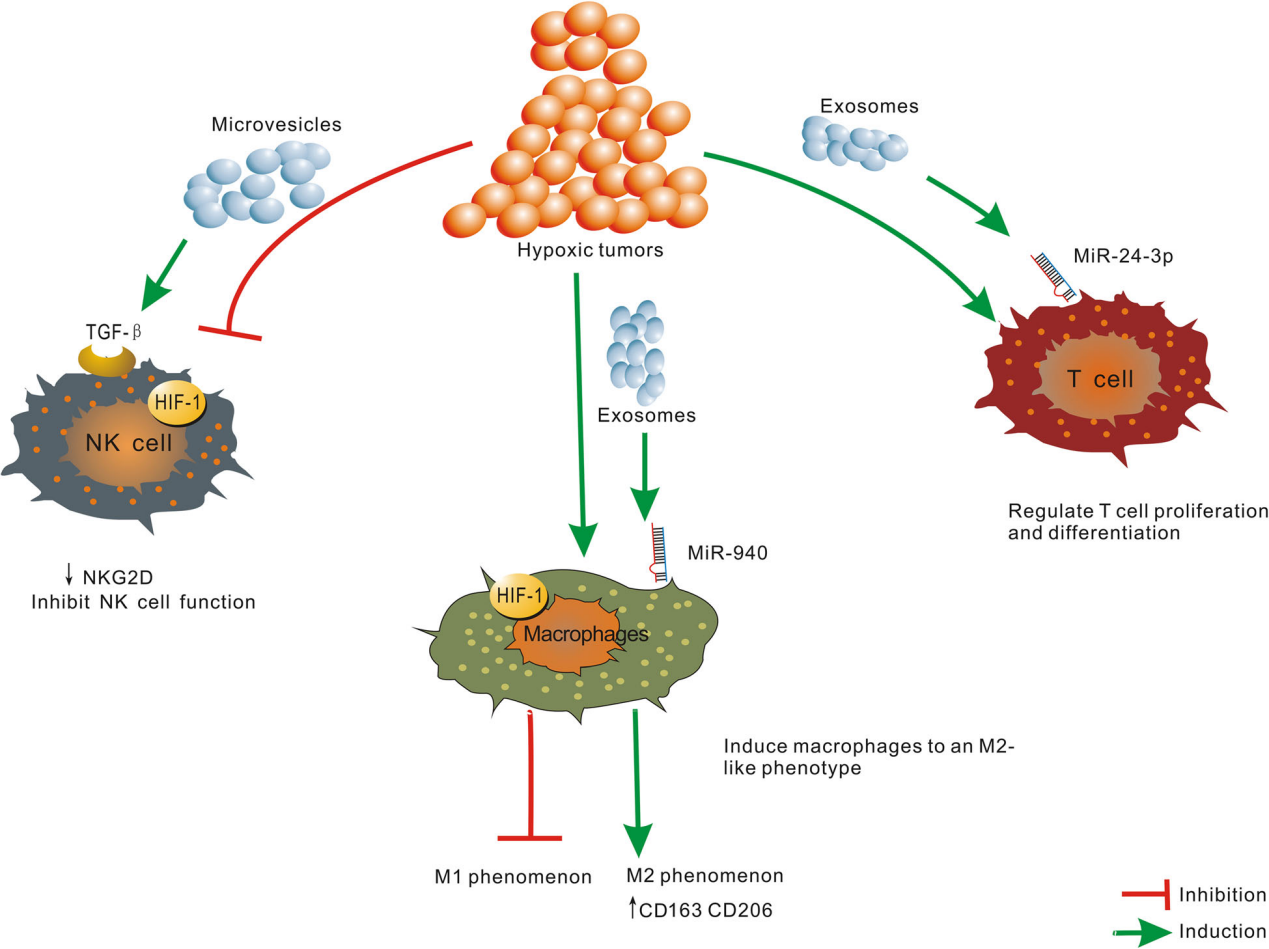
Exosomes and microvesicles derived from hypoxic tumors are involved in the immune response of tumor microenvironment, including regulation of T-cell proliferation, inhibition of NK cells, activation of macrophages, and promotion of their polarization

Interestingly, a recent study found that hypoxia induces macrophage polarization involving the expression of exosomes in epithelial ovarian cancer [70]. In addition, the study demonstrated that SKOv3 cells under hypoxia expressed much more miR-940 than the cells under normoxia, as well as, in exosomes. Moreover, hypoxic exosomes induced macrophages to express higher levels of the M2-type markers, CD163 and CD206, as compared to normoxic exosomes. These data suggested that exosomes could deliver miR-940 to macrophages and that hypoxic exosomes could induce macrophages to an M2-like phenotype. Also, Berchem demonstrated that hypoxic tumor-derived MVs (TD-MVs) negatively regulate the NK cell function by a mechanism involving TGF-β1 and miR23a transfer. The hypoxic TD-MVs transfer TGF-β1 to NK cells, decreasing the cell surface expression of the activating receptor NKG2D, thereby inhibiting the NK cell function. Subsequently, miR-23a in hypoxic TD-MVs serve as an additional immunosuppressive factor as it directly targets the expression of CD107a in NK cells. Moreover, exosomal miR-24-3p is enriched in hypoxic cells from nasopharyngeal carcinoma (NPC) cells. The present study showed that NPC tumor-derived exosomes inhibit T-cell proliferation and Th1 and Th17 differentiation, while inducing the differentiation of regulatory T-cells (Tregs) [71] (Fig. 2).

In conclusion, exosomes act as biologically active vesicles that exert a negative impact on the functions of different types of immune cells by mechanisms engaging more than one molecular pathway responsible for the genetic changes in recipient cells.

### Future perspectives

According to the studies mentioned above, hypoxia-induced exosome-mediated cellular communication is a key signaling mechanism involved in numerous pathological problems. A major mechanism of exosome-mediated cell-to-cell communication is speculated to exert protection of the encapsulated components from degradation, thereby allowing the transfer of exosomal cargo to distant recipient cells. This phenomenon suggests that the biological effects of exosomes are exerted following cellular entry and cargo release. However, the contribution of initial signaling activation upon attachment of exosomes to recipient cells with respect to the functional effects of the subsequent cargo transfer remains unclear. Despite extensive research on the role of specific cargo delivery for exosome-mediated functions, the mechanisms underlying cellular exosomal capture and internalization have received less attention. Thus, understanding the mechanisms of hypoxic exosomes transfer and target cell selection would improve the prospect of therapeutic targeting of exosomes and their development as therapeutic delivery vehicles. Nevertheless, several challenges are noted for future research on hypoxic exosomes. The direct mechanism of induction extracellular vesicles by HIF and its impact in the modulating processes of exosomes formation and release remains to be determined. Also, the orchestration of these processes during hypoxia necessitate further investigation.

## Conclusion

Hypoxia/HIF regulation on exosome and microvesicles function is now recognized as a new and exciting area in cancer research. The discovery of hypoxia-induced exosomes, especially in the context of long-term hypoxia, has led to an intensive focus on tumor angiogenesis, invasion-metastasis, and immune system in various cancer types. Further investigation is warranted for better understanding the alterations in exosome production and release machinery under hypoxic conditions, which would be helpful in targeting the production of exosomes and prevent metastatic onset in the tumor cells.

## Abbreviations

DC: Dendritic cells
ECM: Extracellular matrix
EGFR: Epidermal growth factor receptor
EMT: Epithelial-to-mesenchymal transition
EVs: Extracellular vesicles
FIH: Factor-inhibiting HIF-1α
HIF: Hypoxia-inducible factor
HR-MM: Hypoxia-resistant multiple myeloma.
lncRNAs: Long non-coding RNAs
miRNAs: MicroRNAs
MSDC: Myeloid-derived suppressor cell
MVBs: Multivesicular bodies
NK: Natural killer cells
NPC: Nasopharyngeal carcinoma
PHD: Prolylhydroxylase
TAM: tumor-Associated macrophages
TDEs: Tumor-derived exosomes
TD-MVs: Tumor-derived microvesicles
TGF-LAP: Transforming growth factor-latency associated protein
TIL: Tumor-infiltrating lymphoid cells
TLR: Toll-like receptor
Tregs: Regulatory T-cells
VHL: von Hippel-Lindau
